# Molecular Cloning, Characterization, and Nutritional Regulation of Elovl6 in Large Yellow Croaker (*Larimichthys crocea*)

**DOI:** 10.3390/ijms20071801

**Published:** 2019-04-11

**Authors:** Yongnan Li, Yuning Pang, Xiaojun Xiang, Jianlong Du, Kangsen Mai, Qinghui Ai

**Affiliations:** 1 Key Laboratory of Aquaculture Nutrition and Feed (Ministry of Agriculture) & Key Laboratory of Mariculture (Ministry of Education), Ocean University of China, 5 Yushan Road, Qingdao 266003, China; nanyongli2013@163.com (Y.L.); 15530793072@163.com (Y.P.); xxj_ouc@163.com (X.X.); hddujianlong@163.com (J.D.); kmai@ouc.edu.cn (K.M.); 2 Laboratory for Marine Fisheries Science and Food Production Processes, Qingdao National Laboratory for Marine Science and Technology, 1 Wenhai Road, Qingdao 266237, China

**Keywords:** large yellow croaker, *elovl6*, cloning, nutritional regulation, fatty acid synthesis

## Abstract

Elongation of very long chain fatty acids protein 6 (Elovl6) is a key enzyme in fatty acid synthesis, which participates in converting palmitate (C16:0) to stearate (C18:0). Although studies of Elovl6 have been carried out in mammals, the nutritional regulation of *elovl6* in fish remains poorly understood. In the present study, the cloning and nutritional regulation of *elovl6* were determined in large yellow croaker. Sequence and phylogenetic analysis revealed that the full-length cDNA of *elovl6* was 1360 bp, including an open reading frame of 810 bp encoding a putative protein of 269 amino acid that possesses the characteristic features of Elovl proteins. The transcript level of *elovl6* was significantly increased in the liver of croaker fed the diets with soybean oil (enriched with 18: 2n-6, LA) or linseed oil (enriched with 18: 3n-3, ALA) than that in croaker fed the diet with fish oil (enriched with 20: 5n-3 and 22: 6n-3). Correspondingly, the *elovl6* expression in croaker’s hepatocytes treated with ALA or LA was remarkably increased compared to the controls. Furthermore, the transcription factors including hepatocyte nuclear factor 1α (HNF1α), CCAAT-enhancer-binding protein β (CEBPβ), retinoid X receptor α (RXRα), and cAMP response element-binding protein 1 (CREB1) greatly enhanced promoter activity of *elovl6* in large yellow croaker, and the expression of transcription factors is consistent with the changes of *elovl6* expression in response to fatty acids *in vivo* and *in vitro*. In conclusion, this study revealed that *elovl6* expression in large yellow croaker could be upregulated by dietary ALA or LA via the increased transcriptional expression of transcription factors including *hnf1α*, *cebpβ*, *rxrα*, and *creb1*.

## 1. Introduction

Long chain fatty acids (LCFAs) are predominantly synthesized in the liver by desaturations and elongations, which contributes a special function for normal physiological metabolism [1]. Elongation of very long-chain fatty acids proteins (Elovl), as rate-limiting enzymes, play a crucial role in the elongation step of LCFAs biosynthesis. Elongation of very long chain fatty acids protein 6 (Elovl6) acts to convert palmitate (C16: 0) or palmitoleic acid (C16:1 n-7) to stearate (C18:0) or vaccenic acid (C18:1 n-7) [2,3,4]. It was first discovered in mice [5]. 

Elovl6 is highly expressed in the liver, adipose tissues, and brain [5,6,7], which is related to lipid metabolism and metabolic diseases including hepatic inflammation, insulin resistance, and atherogenesis [6,8,9,10]. Previous studies have demonstrated that loss of Elovl6 function in mice increased the contents of C16:0 and C16:1n-7, and decreased C18:0 and C18:1n-7 contents [9,11,12]. Correspondingly, overexpression of *elovl6* increased synthesis of 18:0 and 18:1n-7 [13]. The recent study in mammals demonstrated that the expression of *elovl6* was altered by dietary lipid supplementation [14]. Furthermore, Elovl6 is regulated directly or indirectly by lipogenic transcription factors including SREBP-1c, carbohydrate response element binding protein (ChREB) and liver X receptor α (LXRα) [6,9,15]. Although many studies of Elovl6 have been carried out in mammals, almost no research on the nutritional regulation of *elovl6* in fish has been carried out, especially in marine fish.

Large yellow croaker (*Larimichthys crocea*), a particularly notable marine fish with high commercial value in southeast China, belongs to Sciaeni-dae family. It has been a useful model for studying fatty acid (FA) metabolism and lipid deposition of fish [16,17,18,19]. This study aimed to characterize the Elovl6 from the large yellow croaker, and to investigate the expression patterns and regulation mechanism of *elovl6* in response to dietary FA. These results may contribute to a further understanding of nutritional regulation and provide the basis for studying the fatty acid synthesis in fish.

## 2. Results

### 2.1. Sequence and Phylogenetic Analysis of Large Yellow Croaker elovl6

**Figure 1:**
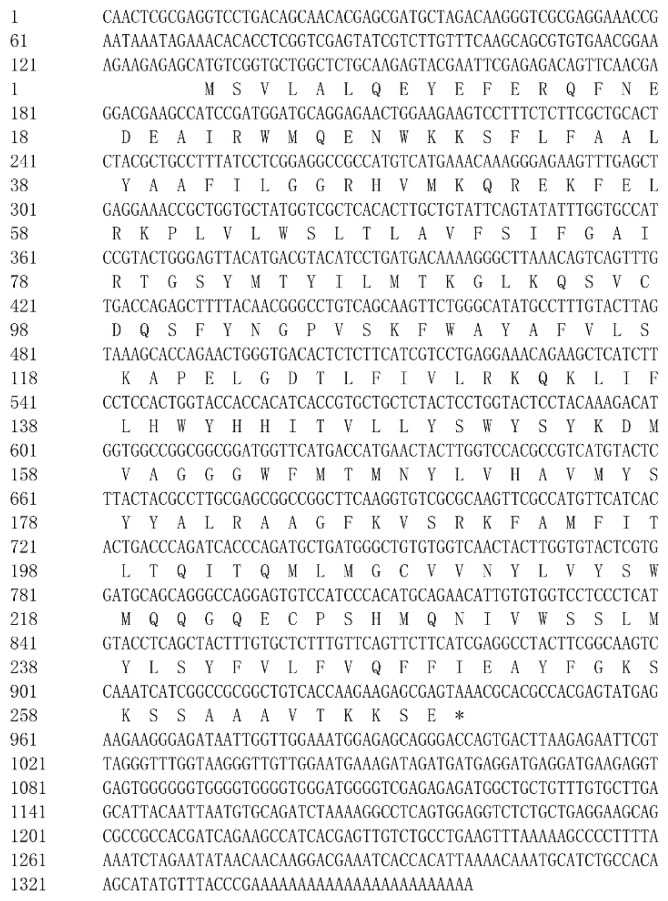
Nucleotide and deduced amino acids sequences of the Elovl6 in large yellow croaker. Uppercase letters indicate the translated region, and lowercase letters indicate the untranslated region.

The full length of large yellow croaker *elovl6* cDNA consisted of 1360 bp that contained an ORF of 810 bp encoding a putative protein of 269 amino acid (AA) (Figure 1). BLAST analysis of deduced amino acid sequence indicated that Elovl6 in large yellow croaker shared high sequence identity with Elovl6 from a variety of teleosts, such as *Lates calcarifer* (97%), *Oreochromis niloticus* (97%), *Salmo salar* (95%), *Oncorhynchus mykiss* (95%), *Cyprinus carpio* (93%), and *Danio rerio* (93%).

**Figure 2:**
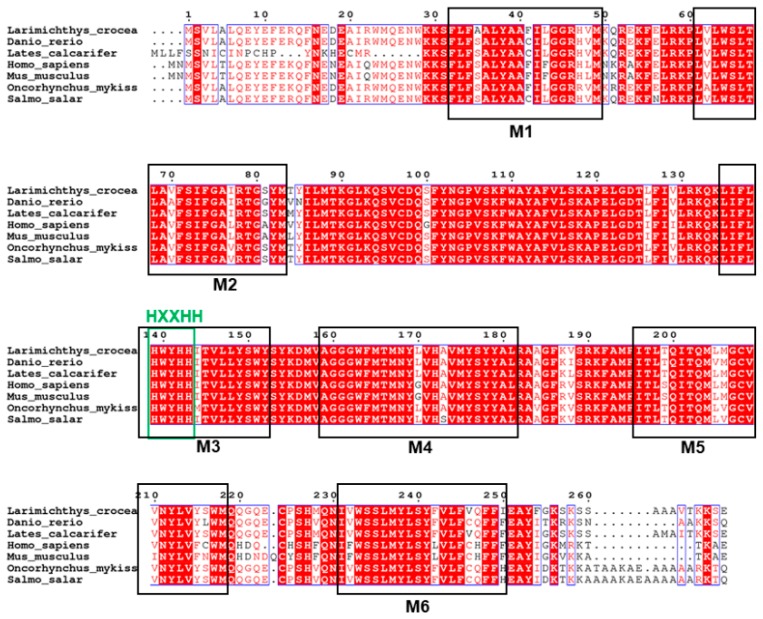
ClustalW amino acid alignment of the deduced amino acid sequence of Elovl6 from large yellow croaker with other fish, mice and humans. The conserved HXXHH histidine box motif and six (M1–M6) putative membrane-spanning domains are indicated.

**Figure 3:**
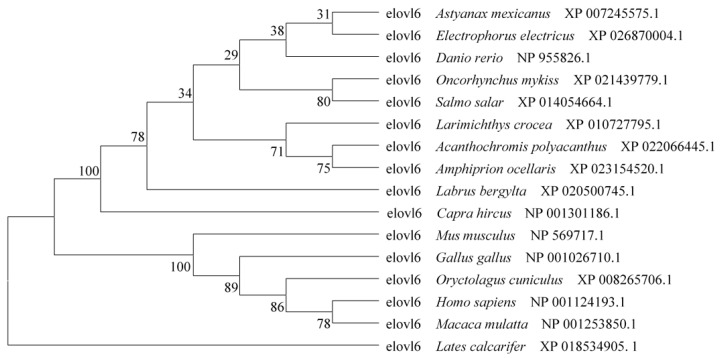
Phylogenetic tree comparing Elovl6 of large yellow croaker with other vertebrate counterparts. The tree was constructed by the neighbor joining method in MEGA4 [20]. The horizontal branch length is proportional to amino acid substitution rate per site. The numbers represent the frequencies (%) with which the tree topology presented was replicated after 1000 iterations.

Sequence analysis of the large yellow croaker Elovl6 proteins showed that possessed the characteristic features of Elovl family members including histidine box (HXXHH) and six membrane-spanning domains (Figure 2). Phylogenetic analysis clustered croaker Elovl6 with several other Elovl6 from other osteichthyes and mammals (Figure 3).

### 2.2. Tissue Distribution of Large Yellow Croaker elovl6

**Figure 4:**
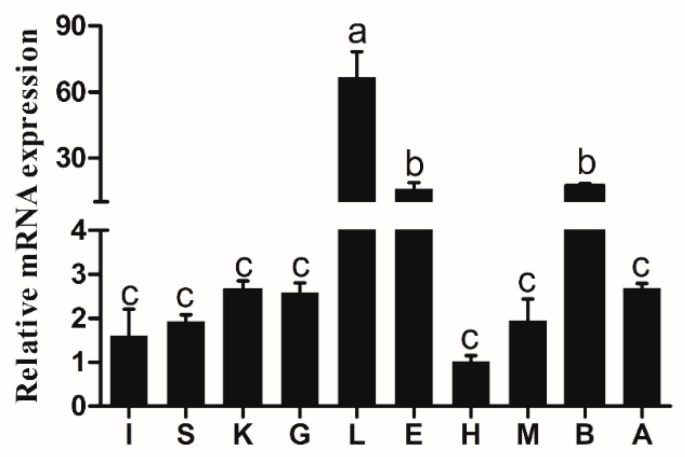
The transcriptional expression of *elovl6* in different tissues of large yellow croaker. intestine (I), spleen (S), kidney (K), gill (G), liver (L), eye (E), heart (H), muscle (M), brain (B), adipose tissue (A). The expression of *elvol6* in the heart was selected as normalization. Data were presented as means ± S.E.M. (*n* = 3). Means share a same superscript letter are not significantly different determined by Tukey’s test (*p* > 0.05).

The transcriptional expression of *elovl6* was determined in different tissues of large yellow croaker fed the diet with fish oil (FO), including the intestine, spleen, kidney, gill, liver, eye, heart, muscle, brain, and adipose tissues. The highest expression of *elovl6* in large yellow croaker was observed in the liver, moderate level of expression was observed in the eye and brain, and the lowest expression was detected in the heart (Figure 4).

### 2.3. Transcriptional Expression of elovl6 in Response to Dietary Fatty Acids

**Figure 5:**
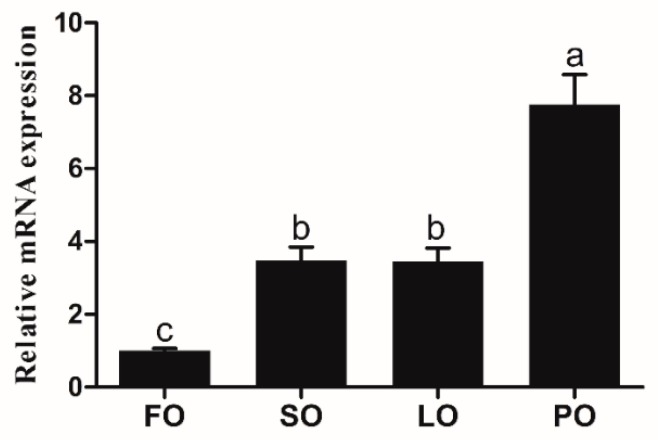
The transcriptional expression of *elovl6* in the liver of large yellow croaker fed the diets with fish oil (FO), soybean oil (SO), linseed oil (LO), or palm oil (PO). The expression of *elvol6* in FO was selected as normalization. Data were presented as means ± S.E.M. (*n* = 3). Means that share the same superscript letter are not significantly different, as determined by Tukey’s test (*p* > 0.05).

The relative mRNA expression of *elovl6* in the liver of large yellow croaker was remarkably affected by dietary fatty acids (*p* < 0.05). The expression of the *elovl6* gene in the liver of large yellow croaker fed the diets with soybean oil (SO), linseed oil (LO), or palm oil (PO) were significantly higher than that in croaker fed the diet with fish oil (FO) (*p* < 0.05), and the highest expression of *elovl6* was observed in PO group (Figure 5).

### 2.4. Relative elovl6 Expression in Hepatocytes of Large Yellow Croaker in Response to Fatty Acids

**Figure 6:**
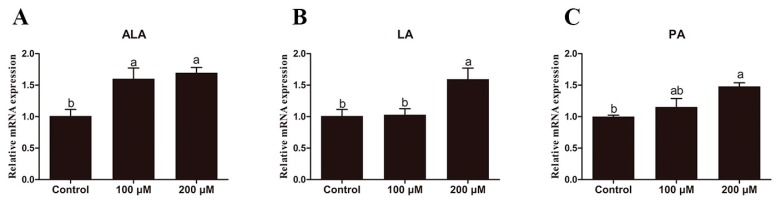
Effects of graded amounts of α-linolenic acid (ALA) and linoleic acid (LA) on *elovl6* expression in hepatocytes of large yellow croaker cultured for 12 h. Large yellow croaker hepatocytes cultured with ALA for 12 h (**A**); Large yellow croaker hepatocytes cultured with LA for 12 h (**B**); Large yellow croaker hepatocytes cultured with PA for 12 h (**C**). The expression of *elvol6* in control group was selected as normalization. Data were presented as means ± S.E.M. (*n* = 3). Means that share the same superscript letter are not significantly different, as determined by Tukey’s test (*p* > 0.05).

Incubation of hepatocytes from large yellow croaker with fatty acids caused distinct changes in *elovl6* expression. Compared with the control group (no supplementation), the relative expression of *elovl6* in hepatocytes from large yellow croaker treated with 100 or 200 μM α-linolenic acid (ALA) for 12 h was significantly increased compared to the control group (*p* < 0.05) (Figure 6A). The expression of *elovl6* in hepatocytes from large yellow croaker treated with 200 μM linoleic acid (LA) and palmitic acid (PA) for 12 h was significantly higher than that of controls (*p* < 0.05) (Figure 6B,C).

### 2.5. Regulation of the elovl6 by Transcription Factors 

**Figure 7:**
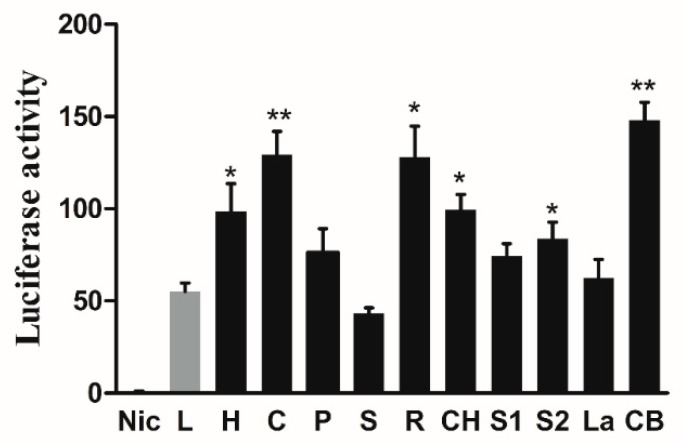
The influence of overexpression of transcription factors on *elovl6* promoter activity in large yellow croaker. The negative control Nic (pGL3-basic) was an empty plasmid with no promoter sequence. The *elovl6* promoter was co-transfected with overexpression plasmids of transcription factor HNF1α (H); CEBPβ (C); PPARγ (P); SP1 (S); RXRα (R); ChREB (CH), SREBP1 (S1); SREBP2 (S2); LXRα (La); and CREB1 (CB), compared with the control group (L) transfected with pGL3- *elovl6* promoter only. The luciferase activity in Nic group was selected as normalization. Data were presented as means ± S.E.M. (*n* = 3). * *p* < 0.05; ** *p* < 0.01.

The promoter of large yellow croaker *elovl6*, an upstream sequence of 2021 bp adjacent to the start of *elovl6* was cloned to characterize the transcriptional regulatory mechanism of *elovl6*. Using bioinformatics software including JASPAR, TRANSFAC, and TF binding, several transcriptional factors binding sites including hepatocyte nuclear factor 1α (HNF1α), CCAAT-enhancer-binding protein β (CEBPβ), peroxisome proliferator-activated receptor γ (PPARγ), transcriptional factor SP1 (SP1), retinoid X receptor α (RXRα), ChREB, SREBP1, SREBP2, LXRα, and cAMP response element-binding protein (CREB1) were predicted within the *elovl6* promoter region of large yellow croaker. The results of dual-luciferase reporter assays showed that the transcription factor HNF1α, CEBPβ, RXRα, ChREB, SREBP2, and CREB1 upregulated the promoter activity of *elovl6* by 1.78-fold, 2.35-fold, 2.32-fold, 1.80-fold, 1.51-fold, and 2.68-fold, respectively. Interestingly, PPARγ, SP1, SREBP1, and LXRα, as important transcription factors in regulation of *elovl6* in mammals, showed no significantly regulatory effect on *elovl6* promoter in large yellow croaker (Figure 7).

**Figure 8:**
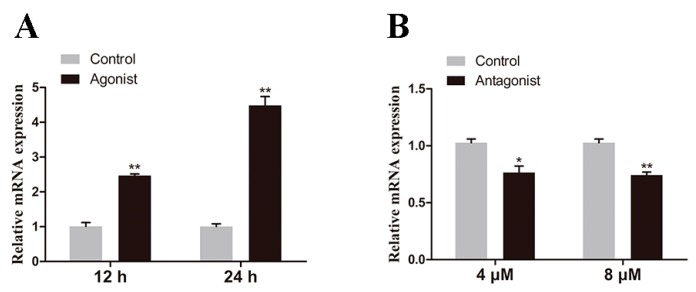
The effects of RXRα agonist (4 μM) (**A**) and antagonist (**B**) on the transcriptional expression of the *elovl6* in hepatocytes of large yellow croaker. The expression of *elovl6* in hepatocytes treated with agonist or antagonist was compared with the control group. The expression of *elvol6* in the control group was selected for normalization. Data were presented as means ± S.E.M. (*n* = 3). * *p* < 0.05; ** *p* < 0.01.

Primary hepatocytes of large yellow croaker were treated with RXRα agonist (Bexarotene) or antagonist (UVI 3003) to verify the regulation of RXRα on *elovl6*. The expression of *elovl6* in hepatocytes incubated with 4 μM RXRα agonist for 12 h or 24 h was up-regulated by 2.46-fold and 4.48-fold, respectively, compared with the control group (*p* < 0.05) (Figure 8A). Furthermore, the expression of *elovl6* in hepatocytes incubated with 4 and 8 μM RXRα antagonist for 1 h was significantly downregulated compared with the control group (*p* < 0.05) (Figure 8B).

### 2.6. Transcriptional Expression of hnf1α, cebpβ, rxrα, and creb1 in Response to Fatty Acids 

**Figure 9:**
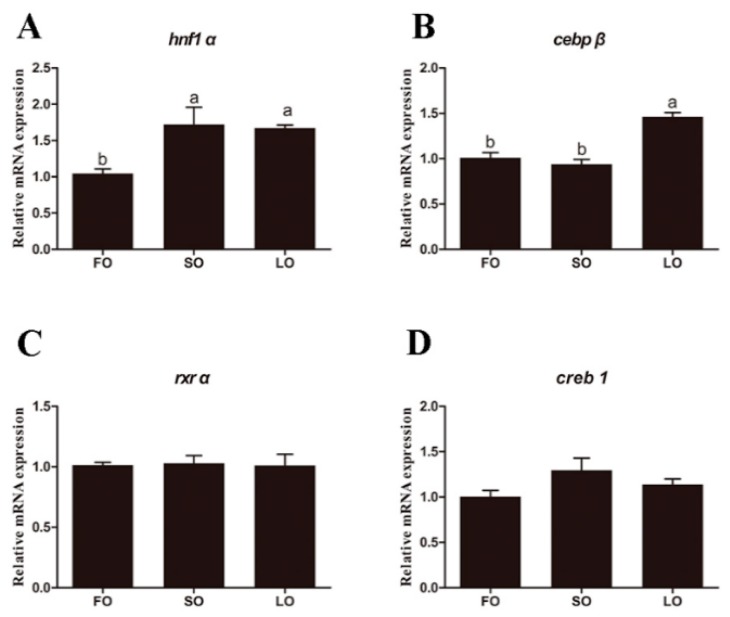
The transcriptional expression of *hnf1α* (**A**), *cebpβ* (**B**), *rxrα* (**C**), and *creb1* (**D**) in the liver of large yellow croaker fed the diets with fish oil (FO), soybean oil (SO), and linseed oil (LO). The expression in FO was selected as normalization. Data were presented as means ± S.E.M. (*n* = 3). Means that share the same superscript letter are not significantly different, as determined by Tukey’s test (*p* > 0.05).

The expression of the *hnf1α* gene in the liver of large yellow croaker fed the diets with SO and LO were significantly higher than that in croaker fed the diet with FO (*p* < 0.05), but there was no significant difference between the SO and LO groups (*p* > 0.05) (Figure 9A). The expression of the *cebpβ* gene in the liver of large yellow croaker fed the diets with LO was significantly higher than that in croaker fed the diet with FO and SO (*p* < 0.05) (Figure 9B). Moreover, the transcriptional expression of *rxrα*, and *creb1* in the liver of large yellow croaker showed no significant difference among three groups (*p* > 0.05) (Figure 9C,D).

**Figure 10:**
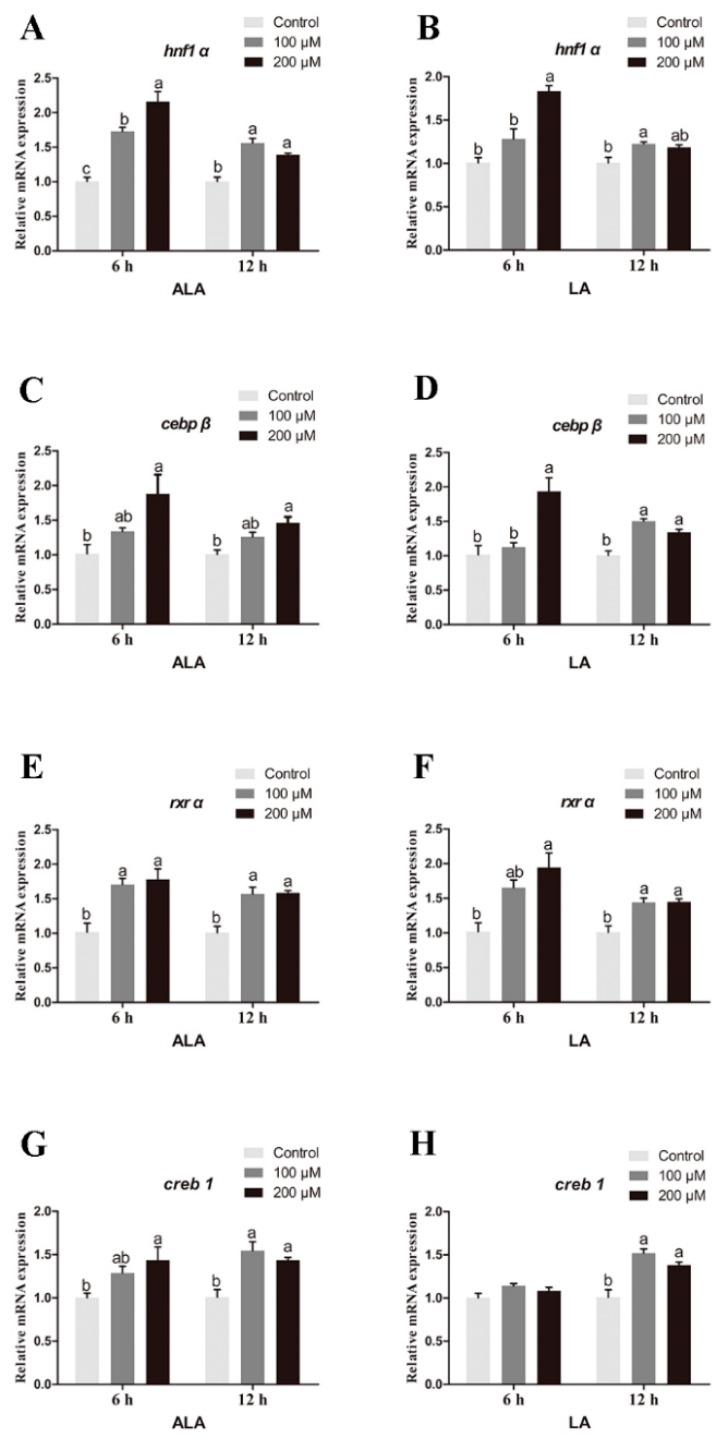
Effects of graded amounts of α-linolenic acid (ALA) and linoleic acid (LA) on *hnf1α*, *cebpb*, *rxrα*, and *creb1* expression in hepatocytes of large yellow croaker cultured for 6 h or 12 h. *hnf1α* with ALA (**A**) or LA (**B**) for 6 h or 12 h; *cebpβ* with ALA (**C**) or LA (**D**); *rxrα* with ALA (**E**) or LA (**F**) for 6 h or 12 h; *creb1* with ALA (G) or LA (H) for 6 h or 12 h. The expression in control group was selected for normalization. Data were presented as means ± S.E.M. (*n* = 3). Means that share the same superscript letter are not significantly different, as determined by Tukey’s test (*p* > 0.05).

Compared with the control group, the relative expression of *hnf1α* in hepatocytes of large yellow croaker treated with 100 or 200 μM ALA for 6 h or 12 h was significantly increased (*p* < 0.05) (Figure 10A). The expression of *hnf1α* in hepatocytes treated with 200 μM LA for 6 h was significantly higher than that of the controls, and the expression in the group of 100 μM LA for 12 h was also higher than that of the controls (*p* < 0.05) (Figure 10B). Compared with the control group, the expression of *cebpβ* in hepatocytes treated with 200 μM ALA for 6 h or 12 h was significantly increased (*p* < 0.05), and the expression in the group of 200 μM LA for 6 h or 12 h was also significantly increased (*p* < 0.05) (Figure 10C,D). The expression of *rxrα* in hepatocytes treated with 100 or 200 μM ALA for 6 h or 12 h was significantly higher than that of the controls (*p* < 0.05), and the expression in the group of 200 μM LA for 6 h or 12 h was also significantly higher than that of the controls (*p* < 0.05) (Figure 10E,F). Compared with the control group, the relative expression of *creb1* in hepatocytes treated with 100 or 200 μM ALA for 6 h or 12 h was significantly increased (*p* < 0.05), and the expression of *creb1* in hepatocytes with 100 or 200 μM LA for 12 h was also significantly increased (*p* < 0.05) (Figure 10G,H).

## 3. Discussion

The Elovl6 protein has been confirmed to catalyze the key step in the elongation of C16: 0 to C18: 0 [7]. Previous studies showed that suppression or deletion of Elovl6 lead to changes in fatty acids composition with decreased stearate (C18:0) and increased palmitate (C16:0) in mice [5,9,10]. However, there is almost no study about Elovl6 in fish. In the present study, the cDNA for *elovl6* gene was cloned from large yellow croaker, and the cDNA exhibited characteristic features of Elovl protein family members, including a single histidine box (HXXHH) and six transmembrane regions [2]. Phylogenetic analysis suggested that deduced amino acid sequence of Elovl6 were highly similar to teleosts and mammals.

In mammals, *elovl6* is highly expressed in lipogenic organs and is sensitive to the diets [6,14,21]. In the present study, the highest expression of *elovl6* in large yellow croaker was observed in the liver, and the relative mRNA expression of *elovl6* was remarkably affected by fatty acids *in vitro* and *in vivo*. The qPCR data for the *elovl6* expression in the liver of croaker fed the diets with PO and hepatocytes treated with PA was significantly higher than that of the controls. PA is the substrate for synthesis of C18:0 and C18:1n-9 as substrate [13]. Hence, the accumulation of PA may promote the transcriptional expression of *elovl6* to drive synthesis of C18:0 and C18:1n-9 using C16:0 as a substrate [3]. Furthermore, the *elovl6* expression in the liver of croaker fed the diets with SO and LO was significantly higher than that in croaker fed the diet with FO. Correspondingly, the *elovl6* expression in hepatocytes treated with LA and ALA was remarkably increased compared to the controls. LA or ALA is the substrate for LC-PUFA synthesis, which is catalyzed by fatty acid desaturases (Fads) and elongation of very long-chain fatty acids (Elovl) [22,23,24,25,26]. Previous studies have shown that transcription factors played crucial role in expression of genes [27]. Thus, it could be speculated that the expression of transcription factors targeting *elovl6* may be increased with the treatment of LA and ALA, which upregulated the transcriptional expression of *elovl6*.

In order to verify the speculation above, *elovl6* promoter luciferase reporter plasmid and the transcription factors expression plasmids were cotransfected into HEK293T cells in the dual-luciferase reporter assay. The studies in mouse suggested that the *elovl6* promoter could be activated by SREBP1c and synergistically activated by both ChREB/Mlx and SREBP1c [7,28]. Furthermore, the transcription factor LXRα could regulate the expression of SREBP1c and ChREB, thus *elovl6* could be regulated by LXRα directly or indirectly [15,29]. In mammals, the expression of *elovl6* could be regulated by the transcription factors including PPARα and PPARγ [3], and SREBP1 and SP1 could increase the bovine *elovl6* promoter activity [30]. However, the transcription factors PPARγ, SP1, SREBP1, and LXRα showed no significant regulatory activity on *elovl6* promoter in large yellow croaker, and only the SREBP2 and ChREB showed activation of *elovl6* promoter activity. The results indicated that the regulation mechanisms of *elovl6* in fish might be different from mammals. It may be correlated with difference in the requirements of essential fatty acids and evolutionary spectacle in two species. Furthermore, transcription factors including HNF1α, CEBPβ, RXRα, and CREB1 could upregulate the activity of *elovl6* promoter in large yellow croaker. Meanwhile, the RXRα agonist (Bexarotene) could greatly increase the transcriptional expression of *elovl6*, and the RXRα antagonist (UVI 3003) suppressed the expression of *elovl6* in large yellow croaker. Thus, the transcription factor RXRα could be a potential target in future studies aiming to regulate the transcriptional expression of *elovl6*. The studies on regulatory effect of these transcription factors (HNF1α, CEBPβ, RXRα, and CREB1) in fish or mammals mainly focused on other genes related to lipid metabolism, with almost no study on *elovl6*, and the finding may contribute to a further understanding of the regulation mechanism of *elovl6*. 

Previous studies have suggested that that *elovl6* could be regulated by dietary long-chain fatty acids (LCFAs) via transcription factors [3,6,28], which is consistent with the findings of the present study. Here, the transcriptional expression of *hnf1α* and *cebpβ* in the liver of large yellow croaker fed the diets with replacement of FO with SO or LO was increased. Correspondingly, the relative expression of *hnf1α*, *cebpβ*, *rxrα*, and *creb1* in hepatocytes treated with LA and ALA was remarkably increased. This is consistent with the changes of *elovl6* expression in response to fatty acids *in vivo* and *in vitro*, and the results suggested that the increase in expression of the transcription factors may be attributed to the increase of *elovl6* expression in croaker treating with ALA or LA. 

In conclusion, the cDNA of *elovl6* was cloned from large yellow croaker and *elovl6* contained the typical features of Elovl proteins. Elovl6 promoter activity was positively regulated by the transcription factors, HNF1α, CEBPβ, RXRα, and CREB1. Furthermore, large yellow croaker treated with LA or ALA resulted in greatly increased expression of *elovl6*, and this increase was likely regulated by transcription factors including HNF1α, CEBPβ, RXRα, and CREB1.

## 4. Materials and Methods

### 4.1. Animal Studies

Four isoproteic (41% crude protein) and isolipidic (12% crude lipid) diets were formulated with the fish oil (FO), soybean oil (SO), linseed oil (LO), and palm oil (PO) as the main lipid sources. The oils purchased from Great Seven Biotechnology Co., Ltd, Qingdao, China and fatty acid profiles of oils were detected (Supplementary Table S1). Large yellow croaker of similar size (mean weight 10.07 ± 0.03 g) from Fu Fa Aquatic Products Co., Ltd., Ningde, China were reared for ten weeks and trail in floating sea cages. Fish were fed with each diet to satiation by hand twice daily at 5:00 and 17:00. At the end of the experiment, samples of intestine, spleen, kidney, gill, liver, eye, heart, muscle, brain, and adipose tissue were collected and snap-frozen in liquid nitrogen after fish fasted for 24 h.

In the present study, all experimental procedures performed on fish and animal care were in strict accordance with the Management Rule of Laboratory Animals (Chinese Order No. 676 of the State Council, revised 1 March 2017).

### 4.2. RNA Extraction and cDNA Synthesis

Total RNA was isolated from the tissues of large yellow croaker using TransZol (TransGen Biotech, Beijing, China) followed by quality measurement on agarose gel electrophoresis and Nanodrop® 2000 (Thermo Fisher Scientific, Waltham, MA, USA). The RNA was reversely transcribed to cDNA by PrimeScriptTM RT reagent kit (Takara, Japan) according to the manufacturer’s instructions.

### 4.3. Cloning, Sequence, and Phylogenetic Analysis of Elovl6

PCR primers of the first fragment for *elovl6* cloning were designed based on sequence (XM_010729493.3). One microliter of liver cDNA, 1 μL of each primer, 12.5 μL of PrimeSTAR® Max DNA Polymerase (Takara, Japan) and 9.5 μL RNA-free water was added in a 25 μL mixture. The PCR program was performed with the following parameters: 35 cycles of 98 °C for 10 s, 55 °C for 15 s and 72 °C for 1 min, followed by 72 °C for 10 min. RACE (rapid amplification of cDNA ends) primers were designed from the sequence of the first *elovl6* fragment. SMARTerTM RACE cDNA Amplification Kit (Clontech, CA, USA) was used to clone the 3′- and 5′-end sequence of *elovl6* according to the manufacturer’s instructions. The PCR program was performed as described above. Then, the PCR products was cloned into pEASY-T1 simple cloning plasmid (TransGen, Beijing, China) for sequencing.

The amino acid (AA) sequence of cloned large yellow croaker *elovl6* cDNA was aligned to orthologues from other vertebrate species. Phylogenetic analysis of the deduced AA sequences of the Elovl6 proteins from large yellow croaker was performed using Mega 4.0. and phylogenetic trees were formed by the neighbor joining method [20].

### 4.4. Relative mRNA Quantification

qPCR of the target genes including *elovl6*, *hnf1α*, *cebpβ*, *rxrα*, and *creb1* was performed in a quantitative thermal cycler (CFX96TM Real-Time System, BIO-RAD, Hercules, CA, USA) by SYBR Green real-time PCR kit (Takara, Japan) according to a previously described procedure [31]. The primer sequences of genes were listed in Table 1. The 2^-ΔΔCT^ method was used to calculate the relative expression of genes [32].

### 4.5. Cloning of elovl6 Promoters from Large Yellow Croaker

The SQ Tissue DNA Kit (OMEGA, Norcross, GA, USA) was used to extracted Genomic DNA from the liver in large yellow croaker according to manufacturer’s instructions. The gene-specific primers for *elovl6* promoter cloning were designed according to the large yellow croaker genomic DNA (National Center of Biotechnology Information, NCBI). The program for *elovl6* promoter cloning was performed by PrimeSTAR® Max DNA Polymerase (Takara, Japan) with the following parameters: 35 cycles of 98 °C for 10 s, 55 °C for 15 s and 72 °C for 1 min, followed by 72 °C for 10 min. Then, the product of PCR was inserted into pGL3-Basic plasmid (Promega, Madison, WI, USA) as a reporter plasmid.

JASPAR (http://jaspar.genereg.net/), TRANSFAC (http://genexplain.com/transfac/) and TF binding (http://tfbind.hgc.jp/) were used to predict potential transcription factor binding sites in the promoter regions of *elovl6* in large yellow croaker.

### 4.6. Construction of Transcription Factor Plasmids, Cell Culturing, Transfection, and Luciferase Assay

For croaker transcription factor plasmids, the ORFs of the HNF1α (GenBank: XM_010754286.3), CEBPβ (GenBank: XM_010741628.3), PPARγ (GenBank: XM_010731330.3), SP1 (GenBank: XM_010732100.3), RXRα (GenBank: XM_019271558.2), ChREB (GenBank: XM_019254372.2), SREBP1(GenBank: KP342262), SREBP2(GenBank: XM_010754025.3), LXRα(GenBank: XM_019273432), and CREB1 (GenBank: XM_027291522.1) were cloned into pCS2^+^ plasmid (Invitrogen, Carlsbad, CA, USA) using ClonExpress II One Step Cloning Kit (Vazyme, Nanjing, China) and primers (Supplemental Table S2).

HEK293T cells were cultured according to a previously described procedure [33]. For transfection, the Elovl6 reporter plasmid (0.2 μg) and transcription factor plasmids (0.6 μg), 20 ng pRL-TK renilla luciferase plasmid, and 2.0 μL Lipofectamine 3000 (Invitrogen, Carlsbad, CA, USA) were co-transfected into the 80% confluence of cells in the 24-well plate in triplicate wells and the luciferase assay was performed by a Dual Luciferase Reporter Assay System (Promega, Madison, WI, USA) followed the manufacturer’s instructions using an InfiniTE200 plate reader (Tecan, Switzerland).

### 4.7. Primary Hepatocyte Isolation and Culture

The isolation and culture of primary hepatocyte from large yellow croaker was performed according to the method described previously [34]. The fatty acids, bovine serum albumin/fatty acid complexes of ALA, LA, and PA (Cayman Chemical Co., Ann Arbor, MI, USA) were supplemented to cells in 6-well plates at a range of concentrations, 100 or 200 μM, for 6 h or 12 h in triplicate wells. The hepatocytes of large yellow croaker were seeded in 6-well plates and incubated with 4 μM of bexarotene (MCE) for 12 and 24 h in triplicate wells, respectively. The hepatocytes of large yellow croaker were also incubated with 4 and 8 μM of UVI 3003 for 1 h in triplicate wells. After incubation, cells were lysed in the wells and harvested for RNA extraction.

### 4.8. Statistical Analysis

The results were presented as means ± S.E.M. The data were analyzed by one-way ANOVA and Student’s *t*-test using SPSS 20.0 with *p* < 0.05 set to be statistically significant.

## Figures and Tables

**Table 1:** Sequences of the primers used in this study for gene cloning, qPCR analysis, and promoter cloning.

Primer	Sequences5′-3′	Primer Information
ORF-F	ATGTCGGTGCTGGCTCTGCAAG	elovl6-ORF
ORF-R	TTACTCGCTCTTCTTGGTGACAGCC	elovl6-ORF
3′-Race-outer	GCCTACTTCGGCAAGTCCAAATCATCGG	elovl6-3′-Race
3′-Race-inner	CCATGAACTACTTGGTCCACGCCGTCATG	elovl6-3′-Race
5′-Race-outer	CGAATTCGTACTCTTGCAGAGCCAGCACCG	elovl6-5′-Race
5′-Race-inner	CTGACAGGCCCGTTGTAAAAGCTCTGGTCAC	elovl6-5′-Race
Elovl6-F	AGGGCTTAAACAGTCAGTTTGTGA	elovl6 q-PCR
Elovl6-R	GAGTAGAGCAGCACGGTGATGTG	elovl6 q-PCR
β-actin-F	CTACGAGGGTTATGCCCTGCC	β-actin q-PCR
β-actin-R	TGAAGGAGTAACCGCGCTCTGT	β-actin q-PCR
RXRα-F	GAGGGACATGCAGATGGATAAGACA	RXRα q-PCR
RXRα-R	GAAGAAGAACAAGTGCTCCAGACACT	RXRα q-PCR
HNF1α-F	AACACGGGGTGAAATACAGC	HNF1α q-PCR
HNF1α-R	GACCAATGAGAAGCGAGGAC	HNF1α q-PCR
CEBPβ-F	GCCCATACCCAAACCCTGAAC	CEBPβ q-PCR
CEBPβ-R	GCACTGTCCTTGCTGATGCTCC	CEBPβ q-PCR
CREB1-F	TCGACTGTTGCTGAGAGCG	CREB1 q-PCR
CREB1-R	GCTGCTGGTCTGATAGATGGG	CREB1 q-PCR
E6-P-F	CATCATCAGACCAGACTGCACAACC	Elovl6 promoter
E6-P-R	GCTCTCTTCTTTCCGTTCACACGC	Elovl6 promoter

## Supplementary Materials

Supplementary materials can be found at https://www.mdpi.com/1422-0067/20/7/1801/s1.

## Abbreviations

AA	amino acid
ALA	α-linolenic acid
CEBPβ	CCAAT-enhancer-binding protein β
ChREB	carbohydrate response element binding protein
CREB1	cAMP response element-binding protein 1
Elovl	Elongation of very long chain fatty acids protein
Elovl6	Elongation of very long chain fatty acids protein 6
FA	fatty acid
Fads	fatty acid desaturases
FO	fish oil
HNF1α	hepatocyte nuclear factor 1α
HXXHH	histidine box
SO	soybean oil
LA	linoleic acid
LCFAs	Long chain fatty acids
LXRα	liver X receptor α
RXRα	retinoid X receptor α
ORF	open reading frame
PO	palm oil
PPARγ	peroxisome proliferator-activated receptor γ
qPCR	Quantitative real-time PCR
SP1	transcriptional factor SP1
SREBP	sterol regulatory element binding protein
LO	linseed oil
PA	palmitic acid
