# Supplementary material for: Molecular Cloning, Characterization, and Nutritional Regulation of Elovl6 in Large Yellow Croaker (Larimichthys crocea)
Source: Int J Mol Sci. 2019 Apr 11;20(7):1801. doi: 10.3390/ijms20071801 (PMC6480403; doi:10.3390/ijms20071801)
Supplement: Supplementary file 1 [file ijms-20-01801-s001.pdf]

Table S1. Fatty acid profiles of palm oil, soybean oil, linseed oil and fish oil (% total fatty acids).

Fatty acid (% total fatty acids)	Palm oil [1]	Soybean oil	Linseed oil	Fish oil
14:0	0.92	0.08	0.07	8.77
16:0	43.01	10.32	5.39	19.15
18:0	3.19	3.95	3.76	4.26
20:0	0.24	0.27	0.17	1.18
16:1n-7	0.25	0.09	0.10	11.85
18:1n-9	30.12	26.10	20.45	9.75
18:2n-6	12.10	49.65	15.52	1.54
20:4n-6	0.00	0.00	0.00	1.30
18:3n-3	0.74	4.92	53.02	0.76
20:5n-3	0.00	0.00	0.00	12.34
22:6n-3	0.00	0.00	0.00	7.30

Reference

1. Li, X.; Ji, R.; Cui, K.; Chen, Q.; Chen, Q.; Fang, W.; Mai, K.; Zhang, Y.; Xu, W.; Ai, Q., High percentage of dietary palm oil suppressed growth and antioxidant capacity and induced the inflammation by activation of TLR-NF-κB signaling pathway in large yellow croaker (Larimichthys crocea). Fish & shellfish immunology 2019, 87, 600–608.

Table S2. Sequences of the primers for construction of transcription factor plasmids.

Primer	Sequences5′-3′
PCS2+-HNF1α-F	CGATTCGAATTCAAGGCCTCTCGAGATGGAGGAGAGGATAGAGGCAGC
PCS2+-HNF1α-R	CTCACTATAGTTCTAGAGGCTCGAGTCACTGAGCCGTAGACACCATCT
PCS2+-CEBPβ-F	CGATTCGAATTCAAGGCCTCTCGAGATGATGTCTGATTCCAGGGTGTC
PCS2+-CEBPβ-R	CTCACTATAGTTCTAGAGGCTCGAGCTAGATATTAGACTCCCCTGCTACTC
PCS2+-PPARγ-F	CGATTCGAATTCAAGGCCTCTCGAGATGCAAACACCAGGCAGAGATT
PCS2+-PPARγ-R	CTCACTATAGTTCTAGAGGCTCGAGCTAATACAAGTCCTTTATGATCTCCTGC
PCS2+-SP1-F	CGATTCGAATTCAAGGCCTCTCGAGATGACCATGGAGGCTGCTGCAGACAC
PCS2+-SP1-R	CTCACTATAGTTCTAGAGGCTCGAGCTACTCTGATTTGTGGCAGTACAAGTC
PCS2+-RXRα-F	CGATTCGAATTCAAGGCCTCTCGAGATGCAACGAGGTCGCTCAAGC
PCS2+-RXRα-R	CTCACTATAGTTCTAGAGGCTCGAGTCATGTCATTTGATGAGGGGCTTCTAGC
PCS2+-ChREB-F	CGATTCGAATTCAAGGCCTCTCGAGATGTACCAGGAGCCTGAGGCTGCGCCGG
PCS2+-ChREB -R	CTCACTATAGTTCTAGAGGCTCGAGTCACATGGGGTGTATGTTGTCCCGGGTGGC
PCS2+-SREBP1-F	CGATTCGAATTCAAGGCCTCTCGAGATGAACAGCCTGTCGTTTGACG
PCS2+-SREBP1-R	CTCACTATAGTTCTAGAGGCTCGAGGCTGTTGGTGACAGTCGTGC
PCS2+-SREBP2-F	CGATTCGAATTCAAGGCCTCTCGAGATGGACGGCGGAGAGTACATCTC
PCS2+-SREBP2-R	CTCACTATAGTTCTAGAGGCTCGAGGGATGCAGCGATGGTCG
PCS2+-LXRα-F	CGATTCGAATTCAAGGCCTCTCGAGATGTCCACGCTGTCTGT
PCS2+-LXRα-R	CTCACTATAGTTCTAGAGGCTCGAGTCACTCGTTGACATCCCAG
PCS2+-CREB1-F	CGATTCGAATTCAAGGCCTCTCGAGATGACCATGGAGGCTGCTGCAGAC
PCS2+-CREB1-R	CTCACTATAGTTCTAGAGGCTCGAGCTACTCTGATTTGTGGCAGTACAAG
